# Frequency, Predictive Factors, and Burden of Gastrointestinal Symptoms in Patients With Type 2 Diabetes

**DOI:** 10.1002/jgh3.70403

**Published:** 2026-04-12

**Authors:** Wah‐Loong Chan, Sarmila Sathiya Seelan, Jeyakantha Ratnasingam, Christine Shamala Selvaraj, Kee‐Huat Chuah, Sanjiv Mahadeva

**Affiliations:** ^1^ Division of Gastroenterology, Department of Medicine, Faculty of Medicine Universiti Malaya Kuala Lumpur Malaysia; ^2^ Department of Internal Medicine Hospital Shah Alam Shah Alam Selangor Malaysia; ^3^ Division of Endocrinology, Department of Medicine, Faculty of Medicine Universiti Malaya Kuala Lumpur Malaysia; ^4^ Department of Primary Care Medicine, Faculty of Medicine Universiti Malaya Kuala Lumpur Malaysia

**Keywords:** Asian, epidemiology, gastrointestinal symptoms, gut‐brain axis, healthcare utilization, type 2 diabetes mellitus

## Abstract

**Aim:**

Gastrointestinal (GI) symptoms are common in diabetes, but their prevalence and impact in Asian adults with type 2 diabetes (T2D) remain unclear.

**Methodology:**

A cross‐sectional study of diabetes patients from a primary care clinic and a diabetes specialized clinic from this institution was conducted. Demographic and clinical data were obtained using validated questionnaires and electronic medical records.

**Results:**

Among 297 patients, 245 (82.5%) reported GI symptoms, 207 (69.7%) had upper GI symptoms and 183 (61.6%) had lower GI symptoms. Anxiety and depression were identified in 11.4% and 8.1% of patients, respectively. Women had a two‐fold increased risk of upper GI symptoms (aOR 2.02; 95% CI 1.22–3.33; *p* = 0.006). Anxiety was linked to upper GI symptoms (88.2% vs. 67.3%; *p* = 0.018), with adjusted analysis showing a threefold increased risk (aOR 3.45; 95% CI 1.17–10.16; *p* = 0.025). Participants older than 50 years had more frequent lower GI symptoms (64.3% vs. 47.9%; *p* = 0.035), with multivariate analysis indicating a trend toward independent risk (aOR 1.92; 95% CI 0.99–3.69; *p* = 0.051). Antiplatelet therapy was also associated with lower GI symptoms (aOR 1.74; 95% CI 1.00–3.01; *p* = 0.047). Diabetes patients with GI symptoms were more likely to undergo upper GI endoscopy (18.8% vs. 9.6%; *p* = 0.07), lower GI endoscopy (13.9% vs. 5.8%; *p* = 0.07), abdominal ultrasound (20.4% vs. 15.4%; *p* = 0.26) and require proton pump inhibitor (22.9% vs. 7.7%; *p* = 0.007).

**Conclusion:**

GI symptoms are very frequent in Asian patients with diabetes, increasing the burden on healthcare utilization. They appear to be influenced by demographic and psychological factors more than the underlying DM per se.

## 1 Introduction

Gastrointestinal (GI)‐related complaints are recognized among patients with diabetes. Glucose control has a close association with GI symptoms, whereby the pathophysiology of GI symptoms in diabetes is complex and multifactorial. Key mechanisms include chronic hyperglycemia‐induced damage, autonomic neuropathy, and impaired GI motility. Gastric and small intestinal motility are highly impaired by hyperglycemia, which later leads to the occurrence of gastroesophageal reflux, heartburn, nausea, vomiting, abdominal bloating, diarrhea, constipation, and so on [1, 2]. In addition, GI motility disorders may give rise to postprandial glycemic dysregulation. Thus, studies have shown that increased frequency of GI disorders, typically in patients with diabetes, negatively influences diabetic control, diabetic complications, diabetic survival, as well as their overall physical and mental well‐being [3, 4].

Both T2D and chronic GI symptoms are common among Malaysian adults. According to a national survey report conducted in 2019, 3.6 million adults (18 years and above) in Malaysia had diabetes, and it is expected to affect ~7 million adults aged 18 and older by 2025, with a diabetes prevalence of 31.3% [5]. On the other hand, chronic GI symptoms such as dyspepsia [6] and irritable bowel syndrome [7] are recognized to affect between 10% and 24% of the adult population as well. However, differences in the epidemiology of both these diseases between the multi‐ethnic population of Malaysia and other regions are recognized [8, 9, 10]. Hence, the nature of the relationship between GI symptoms and T2D in Malaysians may differ from the current published literature.

In this study, we aimed to determine both the frequency and predictive factors of GI symptoms in patients with diabetes. In addition, we explored the burden of GI symptoms in patients with diabetes by collecting data on healthcare utilization for GI symptoms.

## 2 Methodology

### 2.1 Study Design

A cross‐sectional study was carried out in University Malaya Medical Centre (UMMC) between March 2023 and September 2023 involving outpatients from one diabetes specialized clinic and one family physician clinic.

The inclusion criteria for study subjects were as follows: Malaysian citizens, aged 18 years and above, diagnosed with T2D regardless of the duration of the condition, able to understand either English or Malay, and capable of providing written informed consent. The exclusion criteria were as follows: non‐Malaysian citizens, pregnant women, history of peptic ulcer disease, GI malignancy, anatomical obstructions or strictures, inflammatory bowel disease, or coeliac disease.

This study has received approval from the UMMC Medical Research Ethics Committee (MREC ID NO: 2023116‐12001).

### 2.2 Sample Size

Based on an estimated 20% frequency of GI symptoms in adults with T2D [3], we calculated that a sample size of 250 patients was required, with 95% confidence and 5% precision.

### 2.3 Data Collection

Data collection was conducted through a face‐to‐face interview. The questionnaire consisted of basic socio‐demographic information (age, gender, height, weight, ethnicity, educational level, monthly income), lifestyle data (smoking status, alcohol consumption), comorbidities, duration and complications of diabetes, and specific questions on GI symptoms based on the diabetes bowel symptoms questionnaire (DBSQ) [11]. The DBSQ is a 10‐item self‐report screening tool used to assess (upper and lower G) symptoms in patients with diabetes over the past year or months. Questions 1–5 pertain to specific upper GI symptoms such as regurgitation, nausea, meal‐related abdominal discomfort, and so forth. Questions 6–10 pertain to specific lower GI symptoms such as constipation, diarrhea, abdominal pain with defecation, fecal incontinence, and so forth. It uses a 6‐point Likert scale (never to daily). “Never” indicated no symptom, and any other frequencies (from once a month to once a week to daily) were indicative of the presence of symptoms (see Appendix S1).

“Upper GI symptoms” was defined as a “Yes” answer to any of the Questions 1–5.

“Lower GI symptoms” was defined as a “Yes” answer to any of Questions 6–10.

“Any GI symptoms” was defined as responses which included both “Upper” and “Lower” GI symptoms.

Additionally, a locally validated hospital anxiety and depression scale (HADS) [12] was used to assess the severity of anxiety and depression in patients with diabetes who were affected by GI symptoms. Data on biochemical parameters, including fasting blood sugar, HbA1c, fasting lipid profile, renal profile, liver function, and urine albumin creatinine ratio, were obtained from the hospital's electronic medical records. Institutional ethics approval was obtained before the conduct of the study on the 23rd of March 2023.

### 2.4 Statistical Analysis

The data was analyzed using SPSS Statistics software version 20 (IBM, USA). Continuous variables were studied using means, and the means among the continuous variables were compared using a *t*‐test.

Association between two qualitative variables was studied by Pearson's chi square test with *p*‐value less than 0.05 at 95% confidence interval level. Association between the outcomes and exposures was further evaluated using binary logistic regression, with results reported in odd ratios.

## 3 Results

### 3.1 Study Population Characteristics

This study enrolled 297 individuals with T2D, drawn from both primary care clinic (51.2%, *n* = 152) and endocrine clinic (48.8%, *n* = 145). The mean age of 63.4 ± 13.0 years characterizes an older cohort, while the predominance of female participants (57.6%, *n* = 171) and a balanced representation of Malays (34.7%), Chinese (32%), and Indians (33%) reflect Malaysia's multi‐ethnic tapestry. With a mean HbA1c of 8.09% ± 1.94%, it was evident that glycaemic control was generally suboptimal. The lengthy mean diabetes duration of 16.6 ± 11 years underscores the chronicity of the disease in this population. Notably, while 45.8% had no apparent diabetes‐related complications, a substantial proportion, 54.2%, presented with multiple complications. Neuropathy emerged as the most frequent complication (11.4%), followed by atherosclerotic cardiovascular disease (ASCVD) (9.1%) and chronic kidney disease (CKD) (6.1%). Therapeutically, most patients (97%, *n* = 288) received oral antidiabetic agents, while more than half (53.5%, *n* = 159) required insulin. GI complaints were remarkably common, affecting 82.5% of participants. Within this domain, upper GI symptoms were reported by 69.7% and lower GI symptoms by 61.6%. Concurrent psychological assessments indicated that 11.4% experienced anxiety and 8.1% reported depression, suggesting a complex interplay between metabolic, GI, and mental health factors (Table 1).

**TABLE 1:** Basic demography and clinical data.

Location	Family physician clinic = 152 (51.2%)
	Diabetes specialized clinic = 145 (48.8%)
Age (mean ± SD)	63.4 ± 13.0
Gender	Male: 126 (42.4%)
	Female: 171 (57.6%)
Race	Malay: 103 (34.7%)
	Chinese: 95 (32%)
	Indian: 98 (33%)
	Others: 1 (0.3%)
Mean HbA1c (mean ± SD)	8.09 ± 1.94
Duration	
Mean ± SD	16.6 ± 11.0
Median	15.0
Complication	Numerical:
	No complication: 136 (45.8%)
	More than 1 complication: 161 (54.2%)
	Categorical:
	With ASCVD: 27 (9.1%)
	With neuropathy: 34 (11.4%)
	With CKD: 18 (6.1%)
	With retinopathy: 55 (18.5%)
Drugs	Total OGLT: 288 (97%)
	Total insulin: 159 (53.5%)
Upper GI symptoms	With symptoms: 207 (69.7%)
	Without symptoms: 90 (30.3%)
Lower GI symptoms	With symptoms: 183 (61.6%)
	Without symptoms: 114 (38.4%)
Total GI symptoms	With symptoms: 245 (82.5%)
	Without symptoms: 52 (17.5%)
Anxiety	34 (11.4%)
Depression	24 (8.1%)
BMI	BMI < 25: 99 (33.3%)
	BMI ≥ 25: 175 (63.9%)

### 3.2 Predictors of Upper GI Symptoms

Our analyses identified clear demographic and psychosocial determinants of upper GI symptoms among patients with T2D. Female sex and anxiety were associated with upper GI symptoms. Women were more likely than men to report upper GI symptoms (*p* = 0.006), and this remained significant after adjustment (aOR 2.02 [95% CI 1.23–3.33], *p* = 0.006). Participants with anxiety reported more upper GI symptoms than those without anxiety (88.2% vs. 67.3%, *p* = 0.018), and anxiety remained associated after adjustment (aOR 3.45 [95% CI 1.17–10.16], *p* = 0.025). Age, ethnicity, diabetes duration, diabetes complications (including neuropathy and CKD), and antidiabetic therapies (including metformin, GLP‐1 receptor agonists, and insulin) were not significantly associated with upper GI symptoms (Table 2).

**TABLE 2:** Univariate and multivariate analysis of predictors for upper GI symptoms.

Variable	Without GI symptoms	With GI symptoms	Simple logistic regression		Multiple logistic regression *forward stepwise (wald) method was used	
			Crude OR (95% Cl)	*p*	Adjusted OR (95% Cl)	*p*
Age < 50	16 (33.3%)	32 (66.7%)	1.18 (0.61–2.29)	0.618		
Age *≥* 50	74 (29.7%)	175 (70.3%)				
Male	49 (38.9%)	77 (61.1%)	2.02 (1.22–3.33)	0.006	2.02 (1.22–3.33)	0.006
Female	41 (24%)	130 (76.0%)				
Malay	34 (33%)	69 (67%)	1.18 (0.65–2.15)	0.865		
Chinese	28 (29.5%)	67 (70.5%)	1.30 (0.71–2.37)	0.592		
Indian	27 (27.6%)	71 (72.4%)		0.401		
Others	1 (10%)	0 (0.0%)		1.000		
Diabetes specialized clinic	39 (26.9%)	106 (73.1%)	0.73 (0.44–1.20)	0.213		
Family physician clinic	51 (33.6%)	101 (66.4%)				
Duration						
< 15 years	52 (34.0%)	101 (66%)	1.44 (0.87–2.37)	0.16		
*≥* 15 years	38 (26.4%)	106 (73.6%)				
Complications (numerical)						
None	48 (35.3%)	88 (64.7%)	1.55 (0.94–2.54)	0.086		
*≥* 1	42 (26.1%)	119 (73.9%)				
Complications (categorical)						
ASCVD			1.27 (0.52–3.12)	0.604		
No	83 (30.7%)	187 (69.3%)				
Yes	7 (25.9%)	20 (74.1%)				
No neuropathy	83 (31.6%)	180 (68.4%)	1.78 (0.74–4.25)	0.195		
Neuropathy	7 (20.6%)	27 (79.4%)				
No CKD	87 (31.2%)	192 (68.8%)	2.27 (0.64–8.03)	0.205		
CKD	3 (16.7%)	15 (83.3%)				
No metformin	14 (30.4%)	32 (69.6%)	1.01 (0.51–2.00)	0.983		
Yes metformin	76 (30.3%)	175 (69.7%)				
No Insulin	46 (51.1%)	103 (49.8%)	1.08 (0.66–1.77)	0.77		
Yes insulin	44 (48.9%)	104 (50.2%)				
No GLP1RA	82 (31.8%)	176 (68.2%)	1.81 (0.80–4.10)	0.158		
Yes GLP1RA	8 (20.5%)	31 (79.5%)				
No SGLT2i	45 (30.8%)	101 (69.2%)	1.05 (0.64–1.72)	0.848		
Yes SGLT2i	45 (29.8%)	106 (70.2%)				
No DPP4i	70 (31.5%)	152 (68.5%)	1.27 (0.71–2.27)	0.429		
Yes DPP4i	20 (26.7%)	55 (73.3%)				
No sulfonylurea	64 (30.6%)	145 (69.4%)	1.05 (0.61–1.81)	0.854		
Yes sulfonylurea	26 (29.5%)	62 (70.5%)				
No antiplatelets	61 (29.8%)	144 (70.2%)	0.9 (0.54–1.57)	0.760		
Yes antiplatelets	29 (32.2%)	63 (30.4%)				
No anxiety	86 (32.7%)	177 (67.3%)	3.64 (1.24–10.67)	0.018	3.45 (1.17–10.16)	0.025
Yes anxiety	4 (11.8%)	30 (88.2%)				
No depression	87 (31.9%)	186 (68.1%)	3.27 (0.95–11.27)	0.060		
Yes depression	3 (12.5%)	21 (87.5%)				
BMI < 25	27 (27.3%)	72 (72.7%)	0.86 (0.50–1.49)	0.598		
BMI ≥ 25	53 (30.3%)	122 (69.7%)				

*Note:* Grey shade indicates non significance.

### 3.3 Predictors of Lower GI Symptoms

Participants aged > 50 years reported more lower GI symptoms than those aged ≤ 50 years (64.3% vs. 47.9%, *p* = 0.035), with a borderline association after adjustment (aOR 1.92 [95% CI 0.10–3.69], *p* = 0.051). Anxiety was associated with lower GI symptoms and remained significant after adjustment (aOR 2.96 [95% CI 1.21–7.20], *p* = 0.017). Antiplatelet therapy was also associated with lower GI symptoms (aOR 1.74 [95% CI 1.00–3.01], *p* = 0.047). No significant associations were observed for individual complications (including neuropathy and CKD) or for diabetes medications such as GLP‐1 receptor agonists and SGLT2 inhibitors (Table 3).

**TABLE 3:** Univariate and multivariate analysis of predictors for lower GI symptoms.

Variables	Without GI symptoms	With GI symptoms	Simple logistic regression		Multiple logistic regression *forward stepwise (wald) method was used	
			Crude OR (95% Cl)	*p*	Adjusted OR (95% Cl)	*p*
Age < 50	25 (52.1%)	23 (47.9%)	1.95 (1.04–3.64)	0.035	1.92 (0.99–3.69)	0.051
Age *≥* 50	89 (35.7%)	160 (64.3%)				
Male	48 (38.1%)	78 (61.9%)	0.98 (0.610–1.57)	0.930		
Female	66 (38.6%)	105 (61.4%)				
Malay	37 (35.9%)	66 (64.1%)	0.92 (0.52–1.64)	0.858		
Chinese	36 (37.9%)	59 (62.1%)	0.78 (0.44–1.37)	0.774		
Indian	41 (41.8%)	57 (58.2%)		0.390		
Others	0 (0%)	1 (100%)		1.00		
Diabetes specialized clinic	50 (34.5%)	95 (65.5%)	0.72 (0.45–1.16)	0.177		
Family physician clinic	64 (42.1%)	88 (57.9%)				
Duration						
< 15 years	62 (40.5%)	91 (59.5%)	1.21 (0.75–1.93)	0.435		
*≥* 15 years	52 (36.1%)	92 (63.9%)				
Complications (numerical)						
None	62 (45.6%)	74 (54.4%)	1.76 (1.10–2.82)	0.019		
*≥* 1	52 (32.3%)	109 (67.7%)				
Complications (categorical)						
ASCVD			1.88 (0.77–4.59)	0.168		
No	107 (39.6%)	163 (60.4%)				
Yes	7 (25.9%)	20 (74.1%)				
No neuropathy	103 (39.2%)	160 (60.8%)	1.35 (0.63–2.89)	0.443		
Neuropathy	11 (32.4%)	23 (67.6%)				
No CKD	107 (38.4%)	172 (61.6%)	0.98 (0.37–2.60)	0.964		
CKD	7 (38.9%)	11 (61.1%)				
No metformin	15 (32.6%)	31 (67.4%)	0.743 (0.38–1.45)	0.382		
Yes metformin	99 (39.4%)	152 (60.6%)				
No insulin	54 (39.1%)	88 (60.9%)	1.06 (0.65–1.70)	0.805		
Yes insulin	60 (37.7%)	99 (62.3%)				
No GLP1RA	101 (39.1%)	157 (60.9%)	0.78 (0.38–1.58)	0.487		
Yes GLP1RA	13 (33.3%)	26 (66.7%)				
No SGLT2i	55 (37.7%)	91 (62.3%)	0.94 (0.59–1.51)	0.804		
Yes SGLT2i	59 (39.1%)	92 (60.9%)				
No DPP4i	83 (37.4%)	139 (62.6%)	0.85 (0.50–1.45)	0.544		
Yes DPP4i	31 (41.3%)	44 (58.7%)				
No sulfonylurea	81 (38.8%)	128 (61.2%)	1.05 (0.63–1.76)	0.839		
Yes sulfonylurea	33 (37.5%)	55 (62.5%)				
No antiplatelets	88 (42.9%)	117 (57.1%)	1.91 (1.12–3.25)	0.017	1.74 (1.00–3.01)	0.047
Yes antiplatelets	26 (28.3%)	66 (71.7%)				
No anxiety	107 (40.7%)	156 (59.3%)	2.64 (1.11–6.30)	0.028	2.96 (1.21–7.20)	0.017
Yes anxiety	7 (20.6%)	27 (79.4%)				
No depression	107 (39.2%)	166 (60.8%)	1.57 (0.63–3.90)	0.336		
Yes depression	7 (29.2%)	17 (70.8%)				
BMI < 25	39 (39.4%)	60 (60.6%)	1.10 (0.66–1.83)	0.712		
BMI ≥ 25	65 (37.1%)	110 (62.9%)				

*Note:* Grey shade indicates non significance.

### 3.4 Burden of GI Symptoms and Healthcare Utilization

We explored the burden of GI symptoms among adults with T2D, specifically in terms of additional diagnostic procedures and GI‐specific medications. Patients with any GI symptoms were more likely to have an upper GI endoscopy (18.8% vs. 9.6%; *p* = 0.07), lower GI endoscopy (13.9% vs. 5.8%; *p* = 0.07), and abdominal ultrasound imaging (20.4% vs. 15.4%; *p* = 0.26), compared to those without GI symptoms.

**FIGURE 1:**
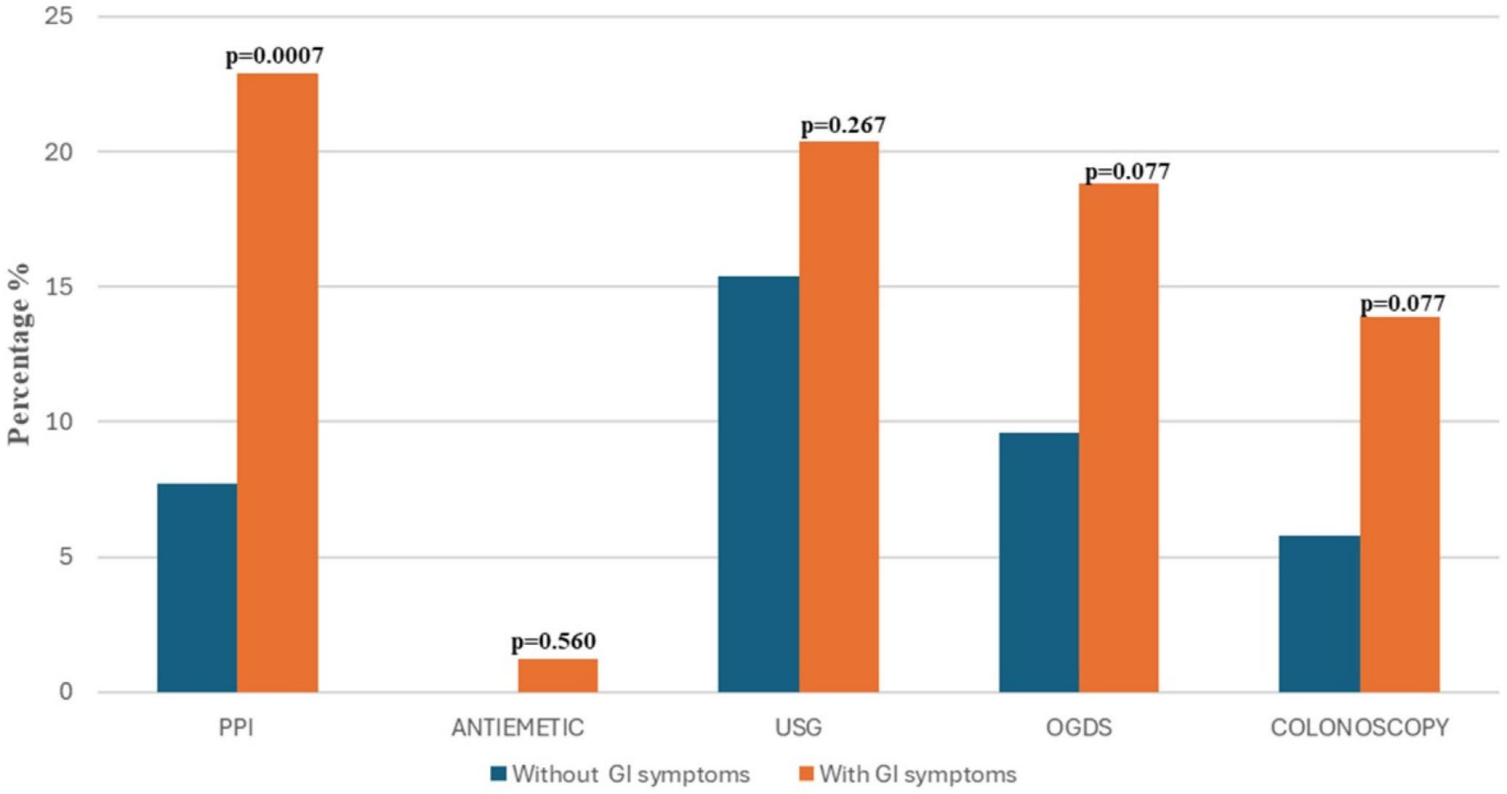
Burden of GI symptoms.

Pharmacologically, patients with diabetes who had GI symptoms were more likely to be prescribed proton pump inhibitors (22.9% vs. 7.7%; *p* = 0.007) (Figure 1).

## 4 Discussion

In this study, 82.5% of patients with T2D reported GI complaints, which is considerably higher than a 52.3% prevalence reported by a recent systematic review and meta‐analysis [13]. One possibility for the high prevalence of GI symptoms may be due to the low symptom frequency threshold used by the DBSQ questionnaire in this study. However, our cohort of DM patients was older with an increased mean age of 63.5 years and patients aged over 50 years reported a higher frequency of lower GI complaints compared to younger individuals (64.3% vs. 47.9%; *p* = 0.033). Although an increased age and anti‐platelet use had a borderline significance for the risk of lower GI symptoms, it is possible that both factors reflected the increased burden of co‐morbid illness commonly associated with GI symptoms [14, 15].

Female gender was independently associated with a twofold increased risk of upper GI symptoms (aOR 2.02; 95% CI [1.22–3.33] L *p* = 0.006). This aligns with findings by Bytzer et al., where GI disturbances were more frequently reported by women, possibly due to differing levels of psychosocial distress, and is further supported by literature indicating a higher prevalence of gastroparesis in females [2, 16, 17]. Such gender‐specific differences highlight the need for a more individualized approach to symptom assessment and management in diabetic populations.

Anxiety was the most consistent independent predictor of GI symptoms in this cohort, with a significant effect on both upper and lower GI domains on multivariate analysis. This parallels previous reports by Talley et al., linking psychological distress to GI dysfunction in diabetics [18]. These observations reinforce the concept of the gut‐brain axis and highlight an urgent need to incorporate mental health assessments into routine diabetes management. Clinicians should therefore avoid attributing GI symptoms exclusively to the underlying metabolic disease or its pharmacotherapy.

In contrast to some reports suggesting that certain oral antidiabetic agents (most notably Metformin [19, 20]) may induce GI side effects, our findings did not support a significant association between any specific oral or injectable antidiabetic medications and GI symptom prevalence. A possible explanation could be that our study was not sufficiently powered to explore the role of Metformin and/or GLP‐1 receptor agonists in causing symptoms. Furthermore, the nature of the study design, which was cross‐sectional, could not identify patients with diabetes who might have discontinued Metformin due to prior symptoms. Nevertheless, our findings are not dissimilar to some other studies, which have demonstrated that bothersome GI symptoms in diabetic patients were not necessarily attributable to their glucose‐lowering therapies [21]. Such reassurance may prove valuable when formulating long‐term management strategies that prioritize glycemia control without disproportionately raising GI concerns.

Diabetes predisposes patients to GI conditions such as gastroparesis, gastroesophageal reflux disease (GERD), and peptic ulcer disease [22, 23] all of which may warrant endoscopic assessment. Additionally, the presentation is often confounded by autonomic neuropathy [24] as well as functional disorders that are prevalent in up to 10%–20% of the general population [25]. This study has reported that patients with diabetes who have GI symptoms are twice as likely to undergo endoscopic examinations in the process of their treatment, adding to the burden of managing these patients. Furthermore, twice to thrice usage of PPIs and anti‐emetics indicates the increased pharmacological burden that GI symptoms add to patients with diabetes.

There are several limitations in our study that warrant consideration when interpreting the results. Firstly, the gender distribution in our study cohort was skewed, with a higher proportion of female participants (57.6%, *n* = 171) compared to males (42.4%, *n* = 126). The female gender has been associated with more GI symptoms in the community, but at a lower frequency than that observed in our study [26]. Secondly, the DBSQ had not been validated in the Malaysian population and the questionnaire may have been misinterpreted by our study subjects. This may have resulted in an over‐estimation of GI symptoms. However, the version of DBSQ used in this study has been validated in other Asian countries, which share a similar demography to our population, and this may have reduced this over‐estimation [16]. Thirdly, the cross‐sectional nature of the study limits the ability to infer causality between T2D, demographic or psychological factors, and the occurrence of GI symptoms. Additionally, as highlighted above, we were not able to identify patients who had previously discontinued metformin due to the development of GI symptoms, potentially underestimating the true prevalence of metformin‐associated GI intolerance.

Fourthly, the absence of a non‐diabetic control group restricts our capacity to delineate the extent to which diabetes itself contributes to the symptom burden. It remains possible that some GI complaints, common in the general population, are merely coincidental with diabetes rather than specifically attributable to it. Nevertheless, we do have historical control data from previous studies of adults with non‐diabetes in primary care, which have reported a prevalence rate of only 10%–24% for GI symptoms [7].

Lastly, the study did not stratify patients based on the severity or duration of their diabetes‐related complications, such as neuropathy, CKD, and ASCVD. These complications, which were present in a subset of our cohort (e.g., neuropathy in 11.4%), are known to exacerbate GI dysfunction through mechanisms like autonomic neuropathy and impaired motility. By failing to account for these factors in detail, the study may have overlooked their potential confounding influence on the reported associations.

## 5 Conclusion

This study underscores the substantial burden of GI symptoms in Asian patients with diabetes. Female gender and anxiety were associated with increased symptom risk, highlighting the importance of psychosocial dimensions in clinical evaluation. Additionally, older age correlated with more pronounced lower GI issues, reflecting the interplay between aging physiology, co‐morbidity, and metabolic disease. Importantly, common antidiabetic medications did not emerge as a key predictor of GI symptoms, suggesting that other demographic and psychological factors warrant greater attention. Moving forward, the integration of mental health assessments, prudent diagnostic resource utilization, and further studies involving control populations will be essential. Such efforts will help refine management strategies, improve patient outcomes, and ensure more holistic care for individuals navigating the complexities of T2D and GI health challenges.

## Funding

The authors have nothing to report.

## Conflicts of Interest

Prof. Sanjiv Mahadeva served as an Associate Editor of JGH Open at the time of submission but was not involved in any editorial decisions related to this manuscript.

## Supporting information


**Appendix S1:** Diabetes bowel symptom questionnaire (DBSQ).

## Data Availability

The data that support the findings of this study are available on request from the corresponding author. The data are not publicly available due to privacy or ethical restrictions.
