# Supplementary material for: Frequency, Predictive Factors, and Burden of Gastrointestinal Symptoms in Patients With Type 2 Diabetes
Source: JGH Open. 2026 Apr 12;10(4):e70403. doi: 10.1002/jgh3.70403 (PMC13070874; doi:10.1002/jgh3.70403)
Supplement: Supplementary file 1 — Appendix S1: Diabetes bowel symptom questionnaire (DBSQ). [file JGH3-10-e70403-s001.docx]

Diabetes Bowel Symptoms Questionnaire (DBSQ) #

| 1. Have you ever experienced heartburn or regurgitation? | 1 Never  2 Less than once a month  3 Once a month  4 Two or three times a month  5 Once a week  6 Several times during the week or everyday |
| --- | --- |
| 1. Have you ever experienced indigestion immediately after meal? | 1 Never  2 Less than once a month  3 Once a month  4 Two or three times a month  5 Once a week  6 Several times during the week or everyday |
| 1. Have you ever experienced nausea and vomiting recently? | 1 Never  2 Less than once a month  3 Once a month  4 Two or three times a month  5 Once a week  6 Several times during the week or everyday |
| 1. Have you ever experienced abdominal bloating or distension? | 1 Never  2 Less than once a month  3 Once a month  4 Two or three times a month  5 Once a week  6 Several times during the week or everyday |
| 1. Have you experienced more than two of the symptom listed below that may suggest gastric ulcers? 2. Abdominal pain relieved after meal or drinking milk 3. Abdominal pain before and after the meal 4. Sleep disturbance due to severe abdominal pain | 1 Never  2 Less than once a month  3 Once a month  4 Two or three times a month  5 Once a week  6 Several times during the week or everyday |
| 1. Have you experienced more than two of the symptom listed below that may suggest irritable bowel syndrome? 2. Pain relieved by defecation 3. Pain occurrence due to frequent defecation or constipation 4. Pain occurrence due to very hard stool or very loose stool | 1 Never  2 Less than once a month  3 Once a month  4 Two or three times a month  5 Once a week  6 Several times during the week or everyday |
| 1. Have you ever experienced diarrhea or loose stool without pain? | 1 Never  2 Less than once a month  3 Once a month  4 Two or three times a month  5 Once a week  6 Several times during the week or everyday |
| 1. Have you ever experienced more than two of the symptoms listed below that might be associated with constipation?   (a)Very hard stool  (b)Defecate less than three times a week  (c)Too much strain on defecation  (d)Residual sensation after defecation  (e) Sensation of anal blockage  (f) Requiring enema for defecation | 1 Never  2 Less than once a month  3 Once a month  4 Two or three times a month  5 Once a week  6 Several times during the week or everyday |
| 1. Have you ever experienced intolerable moderate or severe abdominal pain on the specific area of the abdomen? | 1 Never  2 Less than once a month  3 Once a month  4 Two or three times a month  5 Once a week  6 Several times during the week or everyday |
| 1. Have you ever experienced anal incontinence? | 1 Never  2 Less than once a month  3 Once a month  4 Two or three times a month  5 Once a week  6 Several times during the week or everyday |

# as adapted from Quan C, Talley NJ, Cross S, Jones M, Hammer J, Giles N, Horowitz M. Development and validation of the Diabetes Bowel Symptom Questionnaire. Aliment Pharmacol Ther. 2003;17(9):1179-87.
